# Blood plasma and urinary biomarkers of oxidative stress in cats with urethral obstruction

**DOI:** 10.1186/s12917-024-04009-8

**Published:** 2024-04-27

**Authors:** Fausto Quintavalla, Giuseppina Basini, Francesca Fidanzio, Simona Bussolati, Maria Chiara Sabetti, Maria Cristina Crosta, Stefano Grolli, Roberto Ramoni

**Affiliations:** 1 Department of Veterinary Sciences, University of Parma, Via del Taglio 10, Parma, 43126 Italy; 2 Clinica Veterinaria Gran Sasso of Milan, Milan, Italy

## Abstract

**Background:**

This study aimed to investigate variations of the oxidative status in cats affected by urethral obstruction (UO) under Feline Idiopathic Cystitis (FIC) and Bacterial Cystitis (BC), in comparison with a group of healthy subjects. In both groups, the levels of several markers (either direct or indirect) indicative of the oxidative attack and of the antioxidant response were analyzed on plasma and urine samples. In particular, the plasma samples were evaluated for nitric oxide (NO), hydroperoxides derived by reactive oxygen activity (d-ROMs test), superoxide anion (O_2_^−^), glutathione peroxidase activity (GPx), superoxide dismutase activity (SOD), and ferric reducing antioxidant power (FRAP test); while on urine the levels of NO, d-ROMs, FRAP, SOD, malondialdehyde (MDA) and 8-hydroxydeoxyguanosine (8-OHdG) were measured. Urine of UO patients was also subjected to urine-culture test.

**Results:**

The analytical data on plasma showed that UO, independently of the FIC or BC etiology, induced the insurgence of oxidative stress conditions at the systemic level. In the urine of the UO patients, except for SOD that increased, the markers of redox status were markedly decreased due probably their compromised filtration, thus suggesting involvement of renal function (assessed also by the high levels of plasma creatinine and proteinuria) with no oxidative damage of the lower urinary tract. Moreover, the adoption of a novel oxidative stress index’ (OSI) allowed to establish, by means of a numerical value, the different degrees of oxidative stress conditions for single UO patients, both in terms of oxidative attack and antioxidant response.

**Conclusions:**

Feline urethral obstruction, induced by Idiopathic Cystitis and Bacterial Cystitis, causes oxidative stress conditions at the systemic level that do not interest the lower urinary tract. Despite to the high variability of the profiles of oxidative stress indexes both in healthy and UO patients, the determination of OSI made possible the evaluation of their single degrees of oxidative stress. Possibly the results of this investigation can be compared with those of correspondent pathologies both in humans and in other animal species.

## Background

The finding of urethral obstruction (UO), especially in intact or neutered adult males, is a fairly frequent event in cats with lower urinary tract disease (FLUTD), with incidence of 18–58% [1]. UO may be a functional obstruction secondary to urethral spasm and edema, occurring either alone, or in conjunction with stones or a mucous plug [2]. At least two-thirds of young adult cats (< 10 years of age) affected by FLUTD are diagnosed with feline idiopathic cystitis (FIC), a sterile inflammatory process characterized by intense bladder pain [3–6], nevertheless also cases of bacterial cystitis (BC) are not uncommon [7, 8]. BC can be sporadic or recurrent [8], and the pathogenic bacteria most frequently found in urine are *E. coli*, *Enterococcus fecalis* and *Staphylococcus felis* [9]. In both cases the clinical signs are characterized by periuria, pollakiuria, stranguria and hematuria, but the co-presence of UO represents a potentially life-threatening emergency as patients may be severely compromised, dehydrated, hypovolemic and bradycardic at presentation [10, 11]. It should be underlined that in both FIC and BC the presence of stressful conditions is a common feature [12]. Interestingly, FIC and human interstitial cystitis share many clinical characteristics [13], in fact the terms “idiopathic cystitis” and “interstitial cystitis” are often used interchangeably [14]. For this reason, FIC is often considered the corresponding animal model for bladder pain syndrome in human patients [15], and the evidence that in humans pain symptoms arise from the combination of oxidative damage and free radicals originating from the decrease of blood flow, ischemia, hypoxia and reperfusion, is gradually increasing [16].

Oxidative stress is defined as the imbalance between the formation of reactive oxygen species (ROS) and the antioxidant responses that protect the organism from their chemical aggression. Uncontrolled overproduction of ROS causes protein and lipid peroxidation and DNA damage, that can lead to cell and tissue death [17]. In recent years oxidative stress, that has been studied in various pathological situations of different animal species [18–22], seems to play an important role in the development of their clinical symptoms. Cats have a greater susceptibility to oxidative stress because their antioxidant defenses, especially in the liver and in erythrocytes, are rather limited [23].

In cats, several studies have been focused on the evaluation of oxidative stress in healthy [24, 25] and hospitalized subjects [26] suffering from chronic renal failure [27, 28], infectious peritonitis [29, 30], immunodeficiency virus infection [31], diabetes mellitus [32], hypertrophic cardiomyopathy [17] and hyperthyroidism [33]. All these studies were conducted on blood plasma, evaluating a limited number of parameters. To the authors’ knowledge, the study of oxidative stress on urine samples was limited to the evaluation of F2-isoprostane [34, 35], 8-hydroxydeoxyguanosine (8-OHdG) and malondialdehyde (MDA) [28] in patients with chronic renal disease, and the production of nitric oxide (NO), an inflammatory mediator and a major transmitter that induces urethral relaxation during bladder voiding [36], in the bladder of cats with interstitial cystitis [37]. On this basis, the aim of this work was the evaluation of oxidative stress parameters in both plasma and urine of cats affected by functional urethral obstruction (FIC and BC), compared to clinically healthy cats. In particular, oxidative stress was evaluated in both fluids on the basis of the levels of NO, hydroperoxides (d-ROMs test), superoxide dismutase activity (SOD), and ferric reducing antioxidant power (FRAP test); two other pairs of oxidative stress markers, namely superoxide (O_2_^−^) levels and glutathione peroxidase activity (GPx), malondialdehyde (MDA) and 8-hydroxydeoxyguanosine (8-OHdG) activity, were evaluated only in plasma and in the urine respectively.

## Results

**Fig. 1:**
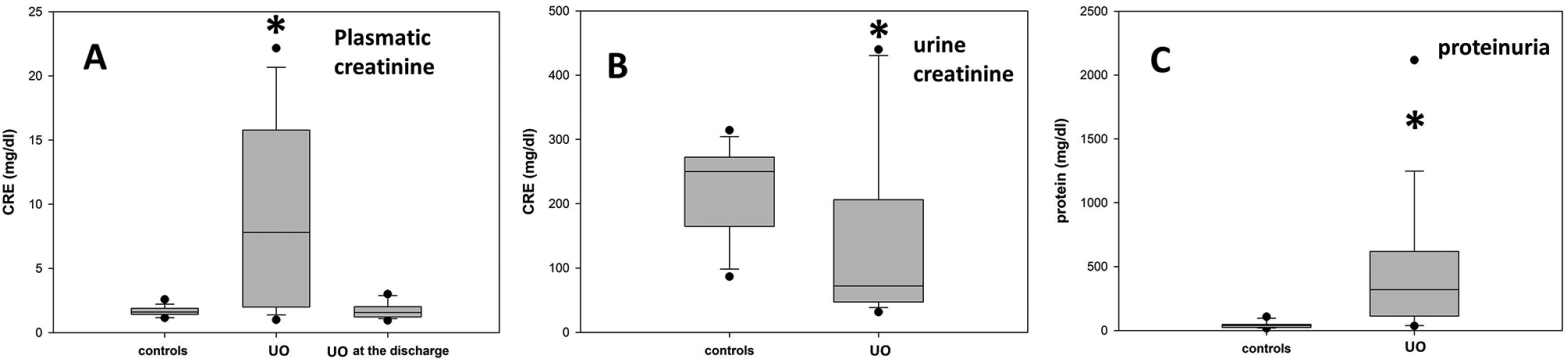
Evaluation of plasmatic creatinine (**A**), urinary creatinine (**B**) and proteinuria (**C**). The graphs report the medians of the different groups; the gray shaded areas include the values considered for the statistical analysis of each group; the asterisks indicate a statistically significant difference between the compared groups, and the dots are the outliers. The error bars indicate the ninetieth percentile of the values for each group of data. A: controls vs. UO patients at the admission *p* < 0.001, UO patients at the admission vs. UO at the discharge *p* < 0.001. B: controls vs. UO patients at the admission *p* = 0.01. D: controls vs. UO patients at the admission *p* = 0.001

**Fig. 2:**
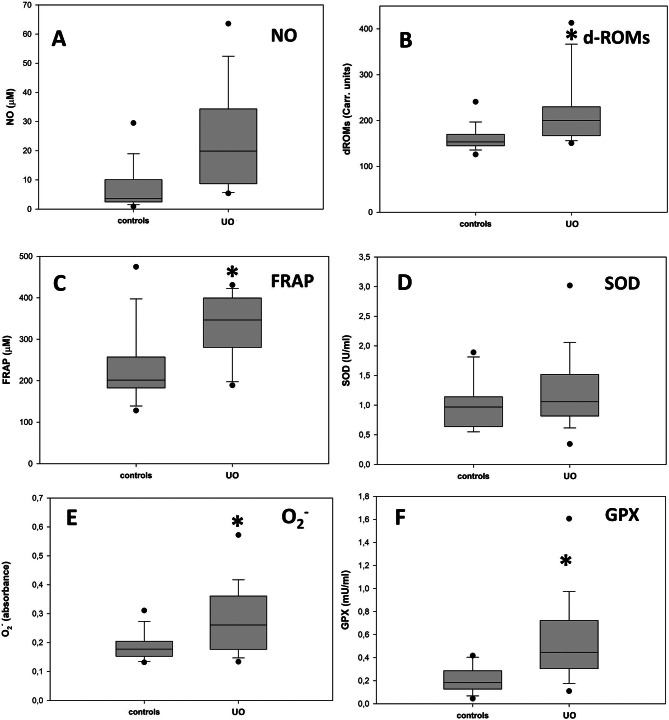
Comparison of the plasmatic levels of different oxidative stress markers between the control group and that of the UO patients. The graphs report the medians of the different groups; the gray shaded areas include the values considered for the statistical analysis of each group; the asterisks indicate a statistically significant difference between the compared groups, and the dots are the outliers. The error bars indicate the ninetieth percentile of the values for each group of data. **A**: Nitric Oxide. **B**: dROMs, *p* < 0.001. C: Ferric Reducing-Antioxidant Power, *p* = 0.001. **D**: Superoxide Dismutase. **E**: Superoxide Anion, *p* = 0.017. **F**: Glutathione Peroxidase, *p* < 0.001

**Fig. 3:**
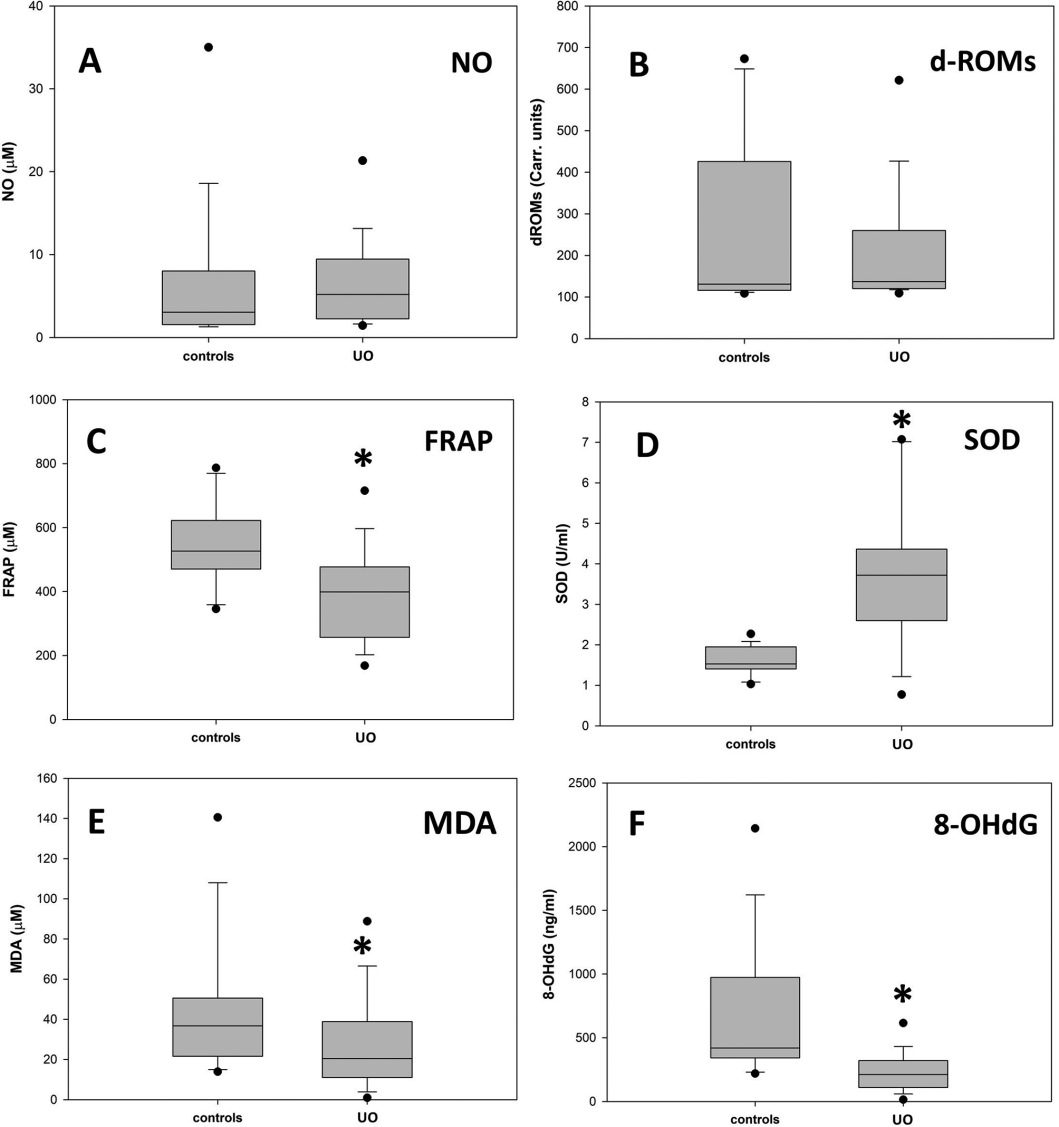
Comparison of the urinary levels d of different oxidative stress markers between the control group and that of the UO patients. The graphs report the medians of the different groups; the gray shaded areas include the values considered for the statistical analysis of each group; the asterisks indicate a statistically significant difference between the compared groups, and the dots are the outliers. The error bars indicate the ninetieth percentile of the values for each group of data. **A**: Nitric Oxide. **B**: dROMs. **C**: Ferric Reducing-Antioxidant Power, *p* = 0.004. **D**: Superoxide Dismutase, *p* < 0.001. E: Malondialdehyde, *p* = 0.042. **F**: 8-hydroxydeoxyguanosine, *p* < 0.001

At the first examination, the blood and urinary biochemical-clinical parameters of all the animals fell within the physiological ranges, except for plasma creatinine (PCr), urine creatinine and proteinuria, that in cats affected by UO (without differences between FIC and BC patients, not shown) differed significantly from those of the control group. PCr levels were higher and distributed over an extremely wide range, urine creatinine was lower, while proteinuria showed a marked increase (Fig. 1). The UO patients of the present study were all subjected to the mechanical removal of the obstruction that determined, for all the subjects, the almost immediate disappearance of the clinical symptoms characterizing this pathology. At the time of the discharge, that occurred after 36 h of observation and without prescription of further pharmacological treatments, in all the UO patients the PCr values ​​had reverted to normal (Fig. 1A). This finding suggests the occurrence, in UO patients, of acute renal distress that was resolved by the removal of the obstruction. All the cats with FIC were sterile on urine culture. In cats belonging to the BC group the following bacteria were isolated in the urine: hemolytic *E. coli* (two patients), *Burkholderia cepacia* (one patient), and *Staphylococcus* sp. hemolytic coagulase negative (three patients). Except for *Staphylococcus* sp, all the bacteria isolated were sensitive to the combination of amoxicillin + clavulanic acid. Nevertheless, due to the rapid disappearance of the clinical symptoms of UO determined by the removal of the obstruction, none of the BC patients was subjected to antibiotic treatment. As regards the markers of oxidative stress, both in plasma and urine of healthy cats and of those affected by UO, they showed great variability, exhibited partial overlaps, and were not normally distributed. Therefore, comparisons between the groups of healthy and pathological cats were assessed using the Mann-Whitney test, which analyzes the significance of the differences between medians. It must be first underlined that since for all the oxidative stress markers it could not be observed, both in plasma and urine, any significant difference related to either gender or the two forms FIC and BC (not shown), the UO patients were considered as a unique group. According to this, the comparisons of the values of the oxidative stress parameters between UO patients and controls are reported in Figs. 2 and 3 for plasma and urine respectively. As shown in Fig. 2, with the only exceptions NO and SOD, statistically significant increases for all the other plasma parameters were assessed between the cats affected by UO and the controls. The oxidative state concerning the plasma of the individual cats was evaluated on the basis of a novel “oxidative stress index” (OSI) which, regardless of the specific units of measurement, collects in a single number, the contribution of the values ​​of the oxidative markers determined in the plasma of each single subject. The numerical contribution N_OX_ of each plasma marker to the OSI for individual cats was calculated from the following ratio:

N_OX_ = measured value of a specific OX marker / median of the values of the same OX marker of the control group.

**Fig. 4:**
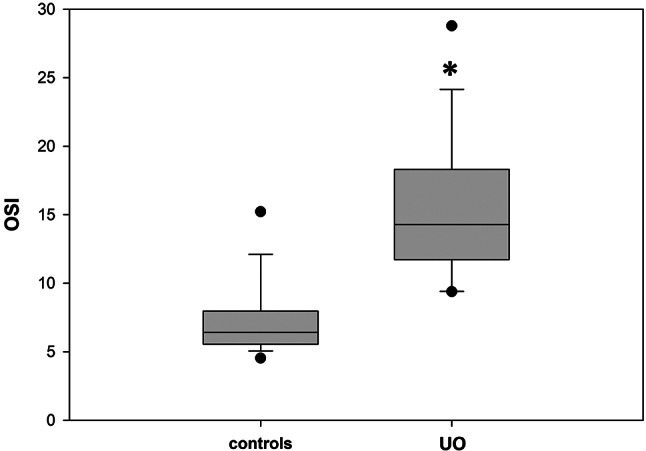
Comparison of the oxidative stress indexes between the controls and the UO patients. The graphs report the medians of the two groups; the gray shaded areas include the values considered for the statistical analysis of each group; the asterisks indicate a statistically significant difference between the compared groups, and the dots are the outliers. The error bars indicate the ninetieth percentile of the values for each group of data. Controls vs. UO *p* < 0.001

**Fig. 5:**
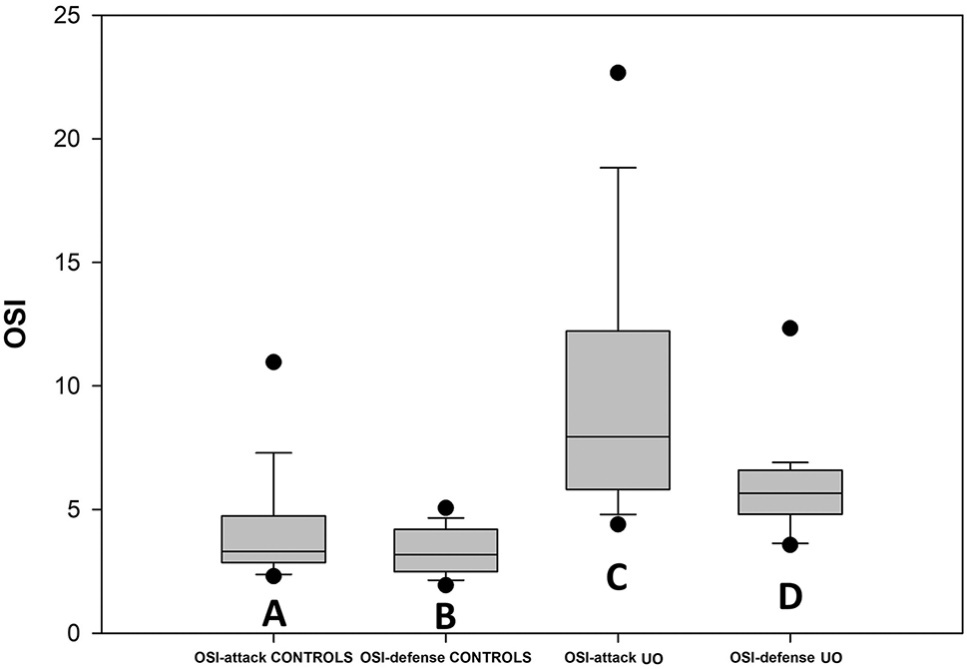
Comparison between the OSI-attack and OSI-defense values of the controls and the UO patients. The graphs report the medians of the groups, the gray shaded areas include the values considered for the statistical analysis of each group, and the dots are the outliers. The error bars indicate the ninetieth percentile of the values for each group of data. Statistically significant differences: A vs. C (*p* < 0.001) and B vs. D (*p* < 0,001)

OSI for a single cat was then determined as the sum of the N_OX_ for all the different oxidation markers considered. The validity of the index as a reliable measure of the oxidative status of each patient is based on the assumption that the medians of the plasmatic markers of the controls can be considered sufficiently reliable as reference values for a population of individuals that are not subjected to oxidative stress. The graph in Fig. 4 reports, as a box plot, the median of the OSI values of plasma of the UO patients in comparison with that of the controls; the error bars indicate the ninetieth percentile of the data of both groups. The two medians are 6,41 and 14,29 for the controls and the UO patients respectively; the zone of overlap between the two data sets occurs for OSI values between 9.42, i.e. the minimum of the UO patients, and 12.00, i.e. the maximum of the controls. To evaluate separately the contributions of the markers measuring biological oxidation from those indicating the antioxidant defense, the OSI value of each subject has been divided into the two following components: OSI-attack, formed as the sum of the N_OX_ values of NO, d-ROMs and O_2_^−^, and OSI-defense, composed by the addition of the N_OX_ values of FRAP, SOD and GPx. The graphs reported in Fig. 5 show that if on one side the OSI-attack (box A) and OSI-defense (box B) values of the controls exhibit, as expected, median values of about 3 (3.3 and 3.2 respectively), on the other UO induces significant increases of both indexes (boxes C and D). Moreover, the graphs of Fig. 5 show that, as it happens for OSI, also the values of the separate OSI-attack and OSI-defense components (A vs. C, and B vs. D) of the UO patients, with the exception of two outliners, do not almost overlap with those of the controls. As regards urine, in Fig. 3 it can be observed that, except for SOD which increased significantly in UO patients, the other oxidative markers either maintained the values of the controls (NO and d-ROMs), or decreased significantly. This behavior, which is in contrast with the observations of plasma markers whose values ​​were generally increased in UO patients, rather than representing the picture of a peculiar oxidative state of the lower urinary tract, probably confirms the presence of a state of acute renal distress, presumably determined by the obstructive event that had been diagnosed on the basis of the levels of proteinuria ​​and of those of plasma and urinary creatinine as shown in Fig. 1. In fact, since SOD is a protein, its increased activity in the urine agrees with the high levels of proteinuria in UO patients shown in Fig. 1; while the decrease of the other markers, which are small molecules, follows the same trend as urinary creatinine, whose decrease in urine is a sign of kidney failure. Therefore, the markers evaluated in urine, whose values are probably influenced by the alteration of the filtration of the excretory organ, do not allow to establish, in UO patients, whether the oxidative state of the lower urinary tract is locally modified by urethral obstruction. Since due to the possible compromission of renal filtration the evaluations of the oxidative status downstream from the kidney are extremely unreliable, OSI and its attack and defense components were not determined for the urine samples.

## Discussion

All the patients affected by UO were castrated males, thus confirming that this pathology is extremely rare in the female gender [38–40]. From the clinical point of view, It must be underlined that immediately after the removal of the urethral obstruction, all the patients restored spontaneous urination, and the symptoms characteristic of this pathology disappeared. Furthermore, after 36 hours of catheterization, all the cats were discharged and returned to their owners, without prescription of further pharmacological treatments. The profiles of the biochemical-clinical tests on plasma and urine at the time of hospital admission did not highlight significant alterations compared to reference values, except for PCr and proteinuria, that were higher compared to those of the controls, and urine creatinine, that was lower, thus suggesting a condition of suffering of the excretory organ. This finding agrees with a previous study where substantial renal and urinary bladder inflammatory lesions were found in cats with UO [41]. The alteration of these parameters, in fact, is indicative of a compromised renal filtration capacity where low molecular weight compounds, like plasma creatinine, are released less efficiently into the urine, and conversely protein macromolecules can pass more easily through the damaged membranes of the renal glomeruli. Although the UO patients were not subjected to a second urine sampling upon discharge, the blood plasma analysis showed, for all of them, a return to normal values ​​of the PCr, that combined with the disappearance of clinical symptoms, suggests a rapid functional recovery of the excretory organ once the urethral obstruction has been removed. Regarding the small group of patients with BC, this study confirms the occasional finding of the disease in cats. Sporadic BC is often due to the frequent presence of comorbidities and exhibits an increased incidence in older cats [8]. The finding here, in a single subject, of *Burkholderia cepacia*, a gram-negative aerobic bacillus whose presence has been rarely detected in the urinary tract of feline BC patients [42, 43], may be of some interest. Since, as indicated above, all the cases were resolved by the prompt removal of the urethral obstruction, the bacteria detected in BC patients, whatever strain they belonged, probably did not have enough time to give rise to infections, classifying these cats, as suggested by some authors, as patients suffering from subclinical bacteriuria disease [44], thus confirming that the empirical administration of antibiotics is not justified in cats with UO [8, 39]. Oxidative stress is a condition caused by the imbalance between the production of pro-oxidants and their elimination through protective antioxidant mechanisms, that in cats have been reported to be more limited, especially in the liver and in erythrocytes [23]. Oxidative stress is a risk factor for lower urinary tract in human patients [36]. ROS can damage detrusor muscle mitochondria, resulting in decreased efficacy of energy production and impaired bladder contraction [45]. Alterations in the redox status have been observed in some animal models of obstructive urinary diseases [46], and it is known that although their effects are unconfirmed in almost all cases, dietary supplements containing polyphenols and other antioxidants can be effective in mitigating the progression of feline cystitis [47]. Considering blood plasma, the values ​​of four of the six parameters considered, i.e. dROMs, FRAP, O_2_^−^ and GPx, in patients affected by UO, were significantly increased compared to those of control cats, while NO and SOD, although not significantly, also showed a tendency towards higher values ​​in the group of pathological cats (Fig. 2). These data indicate that UO causes an increase in oxidative stress (O_2_^−^, d-ROMs and NO) probably at a systemic level, accompanied by the activation of both chemical (FRAP) and enzymatic (SOD and GPx) mechanisms acting to mitigate the harmful effects of biological oxidation. A further possibility is that systemic oxidative stress is determined by the combination of both UO and acute kidney injury. In the urine of both UO patients and controls the oxidative stress condition was assessed by measuring NO, d-ROMs, FRAP and SOD. Furthermore, the contents of MDA and 8-OHdG were evaluated in the urine, that may be related to the onset of lipid peroxidation and oxidative DNA damage in the urinary tract, respectively [48]. In women with bladder pain syndrome, urine concentrations of NO are elevated, and it has also been found to be elevated in FIC cats compared with healthy controls [15, 37, 49], a data that is not confirmed by this study, where NO levels didn’t differ markedly between the group of the UO patients and that of the controls. A possible explanation for this discrepancy could reside in the different method that we adopted for the evaluation of the NO levels. In fact, due to its short half-life, here we employed an indirect test that, although less accurate than the direct assays, allows to evaluate this compound even after a long storage of the samples. With the exception of SOD, whose median was significantly increased compared to that of the controls, the other urinary markers were markedly decreased, suggesting that no oxidative damage occurs in the lower urinary tract of UO patients. On the other hand, obstruction of the urethra caused, as assessed by PCr, a state of acute kidney injury. It is well known that acute kidney injury is caused by multiple pathogenic factors, such as ischemia, nephrotoxic drugs, oxidative stress, inflammation, and urinary tract obstruction [50]. This, in our opinion, would be confirmed by the evidence that low molecular weight compounds such as those detected by the tests for the determinations of NO, FRAP and d-ROMS, whose plasma levels were higher in the UO patients, as a consequence of a compromised renal filtration, were transferred from plasma to urine with a lower efficiency compared to the controls. On the contrary the increased permeability for proteins of the damaged membranes of glomeruli, determines an increase of urinary SOD in the UO patients. Although UO patients are diagnosed with sufferance of the excretory organ, it cannot be excluded that alterations of the metabolic conditions of other organs and tissues eventually determined by variations in hormonal profiles [12, 51, 52], might also contribute to modify their oxidative status. These changes could be determined by alterations of the psychic conditions that in FIC patients may be caused by the persistent pain due to the state of UO. Further hormonal analyzes on plasma samples from UO patients, which have not been performed here, could possibly shed light on this specific point. Since the ranges of values ​​for all parameters are extremely wide, the measurements obtained for a single marker did not allow establish unambiguously the alteration of the oxidative status of individual patient. Therefore, to evaluate the imbalance of the oxidative state of a single subject, a new ‘oxidative stress index’ (OSI) has been adopted here for blood plasma samples. OSI, regardless of the different units of measurement of the various parameters evaluated, considers all the markers measured into a single numerical value. The validity of the index is based on the assumption that the medians of the plasma markers of controls can be considered sufficiently reliable as reference values ​​for a population of individuals not subjected to oxidative stress. Based on the data collected, it can be hypothesized that, with the panel of markers considered for the present investigation, for OSI values ​​higher than the mean value between the median of the controls and that of the UO patients (Fig. 4), i.e. 10.71, single individuals can be considered subjected to a condition of oxidative stress with a probability greater than 90%. It is interesting to observe that, with the exception of only two anomalous values, the overlap zone of OSI between the values of the controls and those of the UO patients (Fig. 4) does not include subjects from both groups, thus confirming the oxidative stress condition of practically all the cats presenting UO. These data suggest that the measurement of a broader panel of plasma markers could lead to the formulation of OSI suitable for more accurate assessments of individual patient conditions in terms of oxidative stress in the case of UO, and possibly for other pathologies. In fact, in principle, higher the number of markers considered, higher should be the difference in the OSI of subjects in conditions of oxidative stress compared to those of controls, even when the variations in the values ​​of some of them would be rather limited. Moreover, by considering separately the N_OX_ values of the markers that measure the oxidative damage and those that evaluate the antioxidant response, OSI has been subdivided into the two ‘attack’ and ‘defense’ components respectively. Interestingly, these two sub-indexes, even with only three markers for the ‘attack’ and ‘defense’ response, have allowed to establish, both on individual and group basis, the efficacy of the antioxidant response determined by the oxidative stress condition induced by UO. These data suggest that a larger panel of both type of markers might aid to better understand the relationship between biological oxidations and the responses that limit their noxious effects, not only in UO patients but also in other pathological conditions.

## Conclusions

The present investigation has made possible to detect that during UO, in cats affected by FIC and BC, an initial state of renal suffering that, if an immediate action is taken to resolve the obstructive event, can be rapidly reverted to normality. The study, that has evaluated the redox state not only on blood but also on urinary samples, has allowed to establish that UO, if promptly diagnosed, doesn’t cause imbalance of the oxidative status of the lower urinary tract. While the evaluation of specific plasma molecular indices has allowed us to establish, for both FIC and BC patients, the onset of systemic oxidative damage, which is counterbalanced by the activation of antioxidant chemical and enzymatic mechanisms. Finally, the determination of novel OSI indexes can represent the basis for more complete investigations on the role of altered biological oxidation in cats affected by UO and possibly in other pathologies with different etiologies.

## Methods

This study was conducted either at the Veterinary Teaching Hospital of the University of Parma (Italy) or at the Clinica Veterinaria Gran Sasso of Milan (Italy). The protocol of this investigation was approved by the institutional ethics committee of the University of Parma ‘OPBA - Organismo preposto al benessere degli animali’ (31/CESA/2021), all methods were carried out in accordance with relevant guidelines and regulations, and cats were prospectively enrolled with the owner consent. The study included a total of 35 cats of different breeds and ages, divided as follows: 19 neutered male cats in the UO group, of which 13 with FIC and 6 with BC, and 16 cats in the control group (5 intact males, 6 neutered males and 5 sterilized females). The cats were of different breeds (UO group: 16 domestic short hair, 1 Norwegian forest; 1 Ragdoll; 1 Scottish fold; Control group: 13 domestic short hair, 2 British shorthair, 1 Ragdoll) and, in both groups the mean age was 6 years. All the cats enrolled lived almost exclusively indoors, with limited access to the exterior environment, and were fed with maintenance dry foods from different manufacturers. All the animals had been vaccinated with core vaccines and periodically treated against internal and external parasites; furthermore, none of the cats had received any medication or antioxidant supplementation starting from 30 days before admission. All the cats were fasting for at least 12 hours before the examination and the collection of blood and urine samples. Inclusion criteria for the UO group were acute clinical signs of urethral obstruction, characterized by straining to urinate without urine output. Urinalysis, including aerobic bacterial culture, radiological and ultrasound examination of the abdomen were performed on all UO patients. These two latest examinations, in particular, allowed to exclude an involvement of an obstructive nature affecting other anatomical structures, and/or to highlight other pathological conditions affecting the urinary system (i.e. hydronephrosis).The maximum duration of the clinical signs before presentation was 48 hours. All these animals, once diagnosed as UO patients, were hospitalized and subjected to a therapeutic treatment following the ISCAID indications, the AAFP/ISFM Guidelines and the AAHA/AAFP 2021 Guidelines [8, 53, 54]. None of the cats, according to the owners, had previously reported episodes of obstructive or non-obstructive FLUTD. Cats were excluded if there were signs of urolithiasis, mineralized urethral plug formation, or neoplasia. The healthy control group consisted of owned cats undergoing annual routine health checks. Cats belonging to this group were included if they had no history of previous urinary tract disease, no clinical signs of urinary tract disease, and if their clinical and laboratory tests were within reference ranges. All subjects had a blood sample taken from the jugular vein and the blood was collected in a tube containing lithium heparin. The plasma samples, obtained by centrifugation at 2,000xg for 10 minutes at room temperature, were divided into two aliquots: one was used immediately for biochemical-clinical investigations, while the remaining part was stored at -80°C until processing (maximum 2 months from collection). The UO patients underwent a second 0.3 ml capillary blood sample to assess plasma creatinine (PCr) levels at discharge. All urine samples were collected by ultrasound-guided cystocentesis, using a 10 ml syringe with a 22Gx1/4” needle. Urine samples were centrifuged immediately after collection at 2,000xg, at 4 °C, for 10 min. The supernatant was separated into two 2 mL aliquots in Eppendorf tubes and one of these was stored at − 80 °C until analysis, while the other 2 ml were used to perform urinalysis. Routine clinical biochemical analysis on plasma and urine samples were performed with an automated chemical analyzer (BT 3500, Biotecnica Instruments, Rome, Italy). Regarding microbiological analysis, urine samples were cultured on Columbia blood agar, MacConkey agar and enrichment broth (Brain Heart Infusion – BHI) for 24 h at 37 °C. After incubation, bacterial growth was assessed, and colonies were isolated and amplified when necessary. Identification of bacterial strains was based on growth and colony characteristics, Gram stain, cell morphology, catalase and oxidase reactions, the API® biochemical test system (bioMérieux, France), and with conventional biochemical tests [55]. The following oxidative stress markers were assessed on the biological samples collected:

- NO - nitric oxide (plasma and urine).
- d-ROMs - derivatives of reactive oxygen metabolites (plasma and urine).
- FRAP - ferric reducing antioxidant power (plasma and urine).
- SOD - superoxide dismutase activity assay (plasma and urine).
- O_2_^−^ - superoxide anion (plasma).
- GPx - glutathione peroxidase assay (plasma).
- 8-OHdG − 8-hydroxydeoxyguanosine (urine).
- MDA - malondialdehyde (urine).

Depending on the markers, they were evaluated either as direct determinations (d-ROMs, 8-OhdG, MDA and O_2_^−^), or on the levels of their byproducts (NO); the enzymes involved in detoxification by oxygen radicals (SOD and GPx) were quantified on the basis of specific activity assays, while the ferric reducing ability of plasma and urine method (FRAP) assessed the capacity of the antioxidants present in the sample to reduce the Fe3^+^/tripyridyltriazine complex under acid pH conditions. Below is reported a description of every assay performed.

### Assay for nitric oxide (NO)

NO was assessed in plasma and urine by measuring nitrite levels with a method based on the formation of a chromophoric compound after reaction with the Griess reagent, that was prepared fresh by mixing equal volumes of stock A (1% sulfanilamide, 5% phosphoric acid) and stock B (0.1% N-[naphthyl] ethylenediamine dihydrochloride in water) solutions. A calibration curve ranging from 0.39 to 25 µΜ was prepared by diluting a water solution of sodium nitrite in distilled H_2_O. The assay was performed in 96-well plates by adding 30 µL of serum to 70 µL of distilled water and 50 µL Griess reagent. After incubation, the absorbance due to the formation of the chromophore was determined with a Victor Nivo colorimeter (Perkin Elmer, Groningen, The Netherlands), by subtracting the value measured at 620 nm to that detected 540 nm.

### Assay for d-ROMs measurement

For the evaluation of hydroperoxides, the d-ROMs test kit (DIACRON International, Grosseto, Italy) was used on both plasma and urine samples. The test is based on the reaction that occurs between hydroperoxides and the iron released from the endogenous proteins in consequence of the acidic pH of the R2 reagent of the kit, that following Fenton reaction mechanism, give rise to peroxyl and alkoxyl radicals (ROOH); these compounds, finally develop a pink color into the R1 reagent of the test, through the reaction with an alkyl-substituted aromatic amine. Briefly, 2 µL of chromogenic substrate (R1) and 200 µL of buffer, pH 4.8 (R2), were mixed with 2 µL of plasma in each well of a microplate. A blank reagent, obtained by replacing the plasma with distilled water and a standard “calibrator” sample, containing known amounts of ROOH (provided by the manufacturer), were included for each assay. After 20 min of incubation at 37 °C, the absorbance was measured at 540 nm by Victor Nivo. The results were expressed in arbitrary units called “Carratelli Units” (CARR U) according to the following formula:

CARR U=[Absorbance sample/Absorbance calibrator]×calibrator.

### Assay for scavenging nonenzymatic activity: ferric reducing-antioxidant power (FRAP)

The FRAP (ferric reducing ability of plasma and urine) method assesses the ability of the antioxidants present in the sample to reduce the Fe3+/tripyridyltriazine complex under acid pH conditions. The reduction of the ferric ion (Fe^3+^) to the ferrous ion (Fe^2+^) takes place according to a colorimetric reaction evaluable by a colorimeter. The reducing ability of the plasma and urine samples was determined by the so called FRAP assay. The FRAP assay measures the change in absorbance at 620 nm due to the formation of a blue colored Fe^2+^-tripyridyltriazine (TPTZ) compound from colorless oxidized Fe^3+^ form by the action of electron donating antioxidants. FRAP reagent was prepared fresh by mixing 25 mL acetate buffer (0.3 M; pH 3.6), 2.5 mL TPTZ (10 mM in 40 mM HCl), and 2.5 mL FeCl3•6H2O (20 mM). Aqueous solutions of known Fe^2+^ (FeSO4•7H2O) concentration in the 100–1000 µM range were used for the calibration curve. The absorbance was recorded with Victor Nivo at 620 nm after a 30-min incubation at 37 °C.

### Assay for superoxide dismutase (SOD)

SOD levels in plasma and urine were assessed by using a commercial enzymatic activity assay (Sigma Chemical Co Lt, St. Louis, MO, USA. The enzyme activity was quantified by measuring the amount of formazan produced by the reaction between tetrazolium salt (WST-1) and superoxide anion (O2−), that is generated by the reaction of an exogenous xanthine oxidase. The remaining O2^−^ is an indirect hint of the endogenous SOD activity. A standard curve of SOD ranging from 0.1 to 200 U/mL was prepared. The color intensity was determined with a Victor Nivo colorimeter by measuring the absorbance at 450 nm, from which was subtracted the absorbance value at 620 nm.

### Assay for superoxide anion (O_2_^−^)

Since evidence exists that tetrazolium salts can be used as a reliable measure of O_2_^−^ production, this parameter was evaluated by the WST-1(Water-soluble tetrazolium salt-1) test (Sigma Chemical Co Lt, St. Louis, MO, USA). The absorbance of each sample was then determined by measuring, with a Victor Nivo colorimeter, the absorbance at 450 nm, from which was subtracted the absorbance value at 620 nm.

### Assay for glutathione peroxidase (GPx)

Glutathione peroxidase is a selenium-containing antioxidant enzyme with the capacity to scavenge free radicals. GPx catalyzes the reduction of various hydroperoxides (e.g., H2O2) to H2O via oxidation of reduced GSH into its disulfide form (GSSG). This enzymatic activity helps to prevent lipid peroxidation and maintain intracellular homeostasis as well as redox balance. Glutathione Peroxidase Assay Kit (Colorimetric) (ab102530) was from Abcam (Cambridge, UK). In the glutathione peroxidase assay protocol, GPx oxidizes GSH to produce GSSG as part of the reaction that reduces hydroperoxide. Glutathione reductase (GR) then reduces the GSSG to produce GSH, and in the same reaction consumes NADPH. The decrease of NADPH (measured as absorbance value at OD = 340 nm by a Victor Nivo colorimeter) is proportional to GPx activity. The assay has a detection sensitivity of ∼ 0.5 mU/ml of GPx in samples.

### Assay for 8-hydroxydeoxyguanosine (8-OHdG)

8-Hydroxydeoxyguanosine is a modified base from DNA that derives from the reaction with hydroxyl radicals that are formed as byproducts and intermediates of aerobic metabolism. Its production is markedly increased during oxidative stress. 8-OHdG amount was determined by competitive ELISA assay ELISA Kit (ab201734; Abcam, Cambridge, UK) that recognizes both free 8-OHdG and DNA-incorporated 8-OHdG. The ELISA utilizes an 8-hydroxy-2-deoxyguanosine-coated plate and an HRP-conjugated antibody for detection. The assay range is 0.94–60 ng/mL. Assay sensitivity is 0.59 ng/mL. Absorbance at 450 nm was read by means a Victor Nivo colorimeter.

### Assay for malondialdehyde (MDA)

MDA, that is an aldehyde generated by lipid peroxidation, is a marker of oxidative stress whose production is markedly increased during urinary infections and diabetic nephropathy [28]. Lipid Peroxidation was here tested by a commercial assay (MDA Assay Kit ab118970; Abcam, Cambridge, UK) following the reaction of the MDA in the sample with thiobarbituric acid (TBA). The MDA-TBA adduct that is generated has been quantified colorimetrically by a Victor Nivo (OD = 532 nm) reader form the absorbance value at 532 nm. This assay detects MDA levels as low as 1 nmol/well.

### Statistical analysis

Results are expressed as means or medians. Differences in means or medians between groups, i.e. controls and UTO patients (BC and FIC patients), and different sex, were compared using ANOVA, Student unpaired t-test or Mann-Whitney rank sum test. Significance was considered when p-values were less than 0.05. All statistical analysis were performed by using the software sigma-plot (Sigma Aldrich - USA).

## Data Availability

The datasets used and/or analysed during the current study are available from the corresponding author on reasonable request.

## Abbreviations

BC: Bacterial Cystitis
CKD: Chronic Kidney Disease
d-ROMs: derivatives of reactive oxygen metabolites
FIC: Feline Idiopathic Cystitis
FLUTD: feline lower urinary tract disease
FRAP: ferric reducing antioxidant power
GPx: glutathione peroxidase
MDA: malondialdehyde
NO: nitric oxide
O_2_^-^: superoxide anion
8-OHdG: 8-hydroxydeoxyguanosine
OSI: oxidative stress index
PCr: plasma creatinine
ROS: reactive oxygen species
SOD: superoxide dismutase activity assay
UO: urethral obstruction
